# Morphology and Physico-Mechanical Threshold of α-Cellulose as Filler in an E-SBR Composite

**DOI:** 10.3390/molecules26030694

**Published:** 2021-01-28

**Authors:** Soumya Ghosh Chowdhury, Jagannath Chanda, Sreedip Ghosh, Abhijit Pal, Prasenjit Ghosh, Sanjay Kumar Bhattacharyya, Rabindra Mukhopadhyay, Shib Shankar Banerjee, Amit Das

**Affiliations:** 1 Hari Shankar Singhania Elastomer and Tyre Research Institute, Plot No. 437, Hebbal Industrial Area, Mysore 570016, Karnataka, India; soumya@jkmail.com (S.G.C.); sreedip.ghosh@jkmail.com (S.G.); abhijit.pal@jkmail.com (A.P.); pghosh@jkmail.com (P.G.); sbhatta@jkmail.com (S.K.B.);rm@jkmail.com (R.M.); 2 Department of Elastomers, Leibniz Institute of Polymer Research Dresden, HoheStraße 6, 01069 Dresden, Germany; shib.pst@gmail.com (S.S.B.); das@ipfdd.de (A.D.)

**Keywords:** natural filler, α-cellulose, E-SBR, reinforcement, physico-mechanical properties, morphology

## Abstract

In the current context of green mobility and sustainability, the use of new generation natural fillers, namely, α-cellulose, has gained significant recognition. The presence of hydroxyl groups on α-cellulose has generated immense eagerness to map its potency as filler in an elastomeric composite. In the present work, α-cellulose-emulsion-grade styrene butadiene rubber (E-SBR) composite is prepared by conventional rubber processing method by using variable proportions of α-cellulose (1 to 40 phr) to assess its reinforce ability. Rheological, physical, visco-elastic and dynamic-mechanical behavior have clearly established that 10 phr loading of α-cellulose can be considered as an optimized dosage in terms of performance parameters. Morphological characterization with the aid of scanning electron microscope (SEM) and transmission electron microscopy (TEM) also substantiated that composite with 10 phr loading of α-cellulose has achieved the morphological threshold. With this background, synthetic filler (silica) is substituted by green filler (α-cellulose) in an E-SBR-based composite. Characterization of the compound has clearly established the reinforcement ability of α-cellulose.

## 1. Introduction

Retrospection of polymer usage in the rubber industry clearly reveals that during the 1930s styrene butadiene rubber (SBR) was developed as a replacement of natural rubber (NR) and rapidly became one of the most common synthetic rubber [1]. SBR is a co-polymer of styrene and 1,3-butadiene. On industrial scale, it is synthesized by two methods, emulsion (E-SBR) and solution (S-SBR) [2] with butadiene to styrene in the proportion (in wt) of 3:1. Micro structurally, butadiene can be observed in cis/trans/vinylic position [2] and formation of micro-structure in E-SBR is un-controllable. As a result, it is formed with broader molecular weight distribution (MWD) and long chain branching. SBR possesses enhanced abrasion resistance, resistance to thermal aging and is lesser prone to mechanical cleavage of chain compared to NR, whereas it has lower resilience, fatigue resistance and lower cut growth resistance with respect to NR [3]. In the rubber industry, E-SBR emerged as one of the majorly used polymers.

Usage of E-SBR would be impossible without the reinforcement of fillers, because reinforcement improves tear and rupture strength of rubber [4]. Till last decade, carbon black (CB) was the sole filler used in different applications due to its better rupture resistance and improved durability [5,6]. Rubber and CB both are hydrophobic, thus mixing of them is generally physi-sorption. In case of silica, strong interaction among surface –OH groups results in poor dispersion which cannot be overcome even by higher mixing energy. Silane coupling agents (SCAs) aid to disperse silica in a rubber matrix through physi-sorption and chemi-sorption mechanisms [7,8,9,10].

In recent times, research on alternative fillers emerging from green sources is gaining importance. Agricultural wastes, like coconut fiber, maize stalks, cherry seed shell, rice husk etc., are explored as fillers by many researchers. For instance, Chigondo et al. modified maize stalks by chemical means and investigated its reinforcement effect in NR composites [11]. Razif et al. examined the impact of rice husk on different mechanical properties of polypropylene composites [12]. Similarly, the footprint of modified linen fiber waste on physico-mechanical properties of polar and non-polar rubber was explored by Hussain et al. [13]. Jacob et al. found that modified sisal fiber imparts superior mechanical properties of NR compounds [14]. Osabohien et al. reported the effect of cherry seed shell as filler in NR composites [15]. Egwaikide et al. explored the effect of coconut fiber as filler on the rheological, physico-mechanical properties of a NR vulcanizate [16].

**Figure 1:**
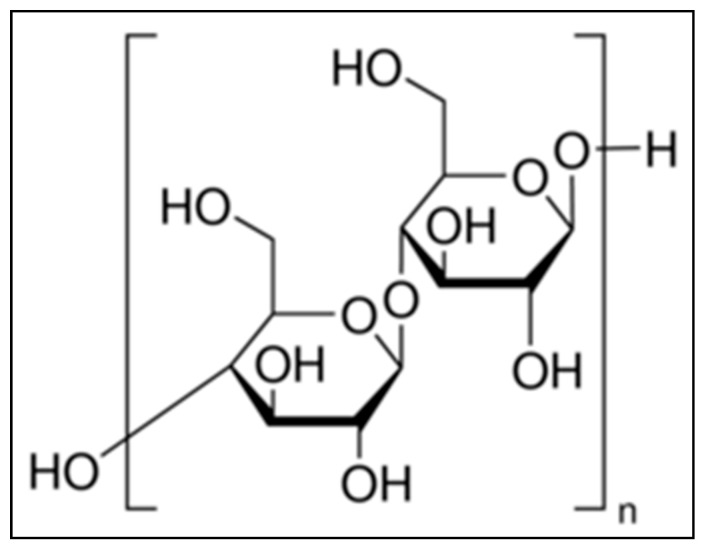
Chemical structure of α-cellulose.

Cellulose, a complex carbohydrate or polysaccharide, is the most abundant naturally occurring organic compound, having a molecular formula of (C_6_H_10_O_5_)_n_ [17,18]. It produces a 95–96% yield of D-glucose unit after hydrolysis with fuming hydrochloric acid. Its structure is based on a D-glucose unit linked by β-1, 4 glycoside bonds. Cellulose comprises about 33% of all vegetable matters. It is the basic structural component of plant cell walls. The chemical structure of α-cellulose is mentioned in Figure 1. Fifty percent of wood and 90% of cotton consists of cellulose. α-cellulose, one of the three classes of cellulose, has the highest degree of polymerization and stability. The other two classes of cellulose are β-cellulose and γ-cellulose. Cellulose has both amorphous and crystalline regions [18]. Removal of the amorphous region by acid hydrolysis increases the strength and stiffness of cellulose. This is due to the presence of only the crystalline region, that helps α-cellulose to act as filler [18].

In this article, potency of α-cellulose as filler and the volume percentage at which optimal reinforcement occurs in the E-SBR matrix is investigated. Fourier transform infrared spectroscopy (FTIR) and scanning electron microscope (SEM) are employed for detailed morphological characterization of α-cellulose. Thermal reactivity as well as degree of purity is measured by thermo-gravimetric analysis (TGA) and differential scanning calorimeter (DSC). Physico-mechanical properties, visco-elastic behavior along with flow characteristics are scrutinized. Scanning electron microscope (SEM) and transmission electron microscopy (TEM) are employed to understand the micro-dispersion of filler at different filler volume fractions. Physico-mechanical and morphological characterizations of the composites are compiled to assess reinforcement behavior as well as optimal volume threshold of α-cellulose (Figure 1) in the E-SBR–α-cellulose composite. Finally, substitution of silica by α-cellulose (10 parts) and subsequent evaluation of mechanical properties establish its reinforcing ability in E-SBR composite.

## 2. Results and Discussion 

### 2.1. Characterization of α-Cellulose

**Figure 2:**
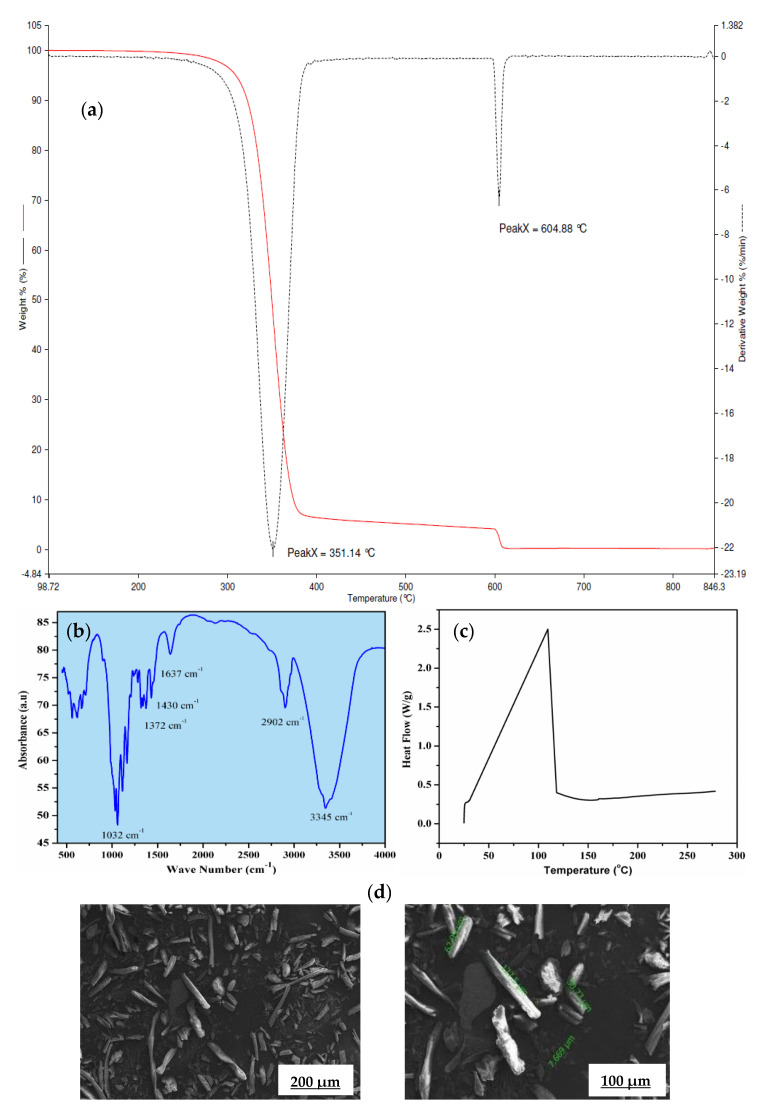
(**a**) Thermo-gravimetric (TG) and differential thermo-gravimetric (DTG) profile of α-cellulose, (**b**) Fourier transform infrared (FTIR) spectrum of α-cellulose, (**c**) heat flow curve of α-cellulose, (**d**) scanning electron microscopic (SEM) images of α-cellulose at 500× and 1000× magnification.

The thermo-gravimetric (TG) and differential thermo-gravimetric (DTG) profile of α-cellulose is shown in Figure 2a. It shows double-stage degradation at 350 and 604 °C, respectively. The first stage is due to loss of bound moisture and the second stage is associated with the main chain degradation. This main chain degradation at very higher temperature suggests high thermal stability of α-cellulose and ensures minimum defection during mixing and consequent processing. Fourier transform infrared spectroscopy (FTIR) is used to characterize structure and chemical composition of α-cellulose [17]. An FTIR spectrum of α-cellulose is presented in Figure 2b.

Absorbance maxima at 3345 cm^−1^ is the characteristic feature of -OH stretching vibration, whereas the band around 2902 cm^−1^ is attributed to the symmetric and asymmetric stretching vibration of C-H bonds present in -CH_2_ of aliphatic chains and end –CH_3_ groups. The signal at 1637 cm^−1^ can be associated with the C=O stretching vibration. Distinct transmittance at 1430 cm^−1^ confirms H-C-H and -O-C-H in-plane bending vibration, whereas in-phase -OH bending vibration results in transmittance at 1372 cm^−1^. Peak at 1032 cm^−1^ may be contributed by C-C, C-OH, C-H ring and side group vibration. Presence of hydroxyl (-OH) groups in α-cellulose is the key structural feature that has created enthusiasm to map its potency as a reinforcing filler in the E-SBR matrix.

Thermal reactivity of α-cellulose is studied by using differential scanning calorimeter, Figure 2c. An endothermic peak was observed at 85 °C, which corresponds to the loss of moisture. The experiment is conducted up to 280 °C. No physical change is observed up to heating at that temperature (at a heating rate of 10 °C/min.). In accordance with the fact, the moisture loss at 85 °C has not caused any cure defects while preparing tensile slabs for the α-cellulose compounds.

α-cellulose exhibits small aggregates due to the presence of strong Van der Waals interaction between surface hydroxyl groups. In addition to that, it also revealed that the presence of individualized and crystalline structures with L/D ratio is more than 1. Some tubular structures are also observed at higher magnification with length value ranging from 7 to 127 μm (Figure 2d). This typical dimension of α-cellulose generates keenness to understand its potential as filler in a rubber matrix. Absence of strong aggregates is expected to facilitate easy dispersion and distribution of α-cellulose particles in rubber matrix. The compositions of the α-cellulose containing rubber compounds are shown in Table 1 where the amount α-cellulose is increased progressively in an E-SBR matrix.

### 2.2. Characterization of E-SBR-α-Cellulose Composites

#### 2.2.1. Investigation on Morphological Threshold

**Figure 3:**
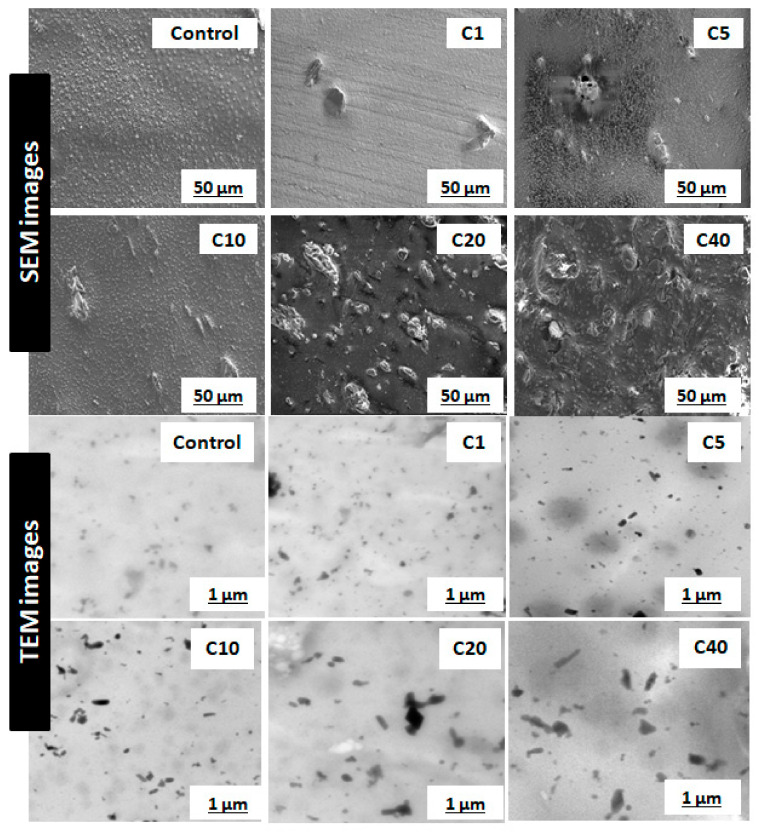
Scanning electron microscopy (SEM) images of cured stocks at 2500× magnification; transmission electron microscopy (TEM) images of cured stocks at 8600× magnification.

The most critical parameter for evaluation of α-cellulose is its dispersibility and distribution in the polymeric matrix. Characterization of filler morphology ranging from micro- to macro-scale determines reinforcing ability of α-cellulose. Microscopic analysis involving different instruments, namely SEM and TEM, are important to understand spatial resolution and orientation of filler particles throughout the matrix. SEM and TEM images of cured compounds are shown in Figure 3. It represents dispersion as well as distribution of α-cellulose in the elastomeric matrix. The images represented here are from cured stocks and captured at 2500× magnification for SEM and at 8600× magnification in the case of TEM. From TEM images, in the case of C1, C5 and C10, α-cellulose is well distributed throughout the E-SBR matrix. The increased dosage of filler (α-cellulose) leads to re-agglomeration of α-cellulose, which is very much evident from TEM images (Figure 3). Representative images of C20 and C40 clearly demonstrates agglomerated sites in the polymer matrix.

SEM images also designated C10 as the morphological threshold amongst all. Non-uniformly distributed large clusters of α-cellulose are visible in higher dosage compounds, namely C20 and C40. These aggregated sites can induce flaw within the matrix. Therefore, the SEM images confer that 10 phr dosage of α-cellulose is the optimum one with regard to dispersion in the emulsion-grade SBR matrix.

#### 2.2.2. Filler Dispersion and Filler Networking

Introduction of filler within an unfilled elastomeric matrix is a conglomerate phenomenon. Hydrodynamic reinforcement provided by filler particles can be predicted by Guth and Gold equation when unfilled elastomers get reinforced [19]. An increase in the filler volume fraction instigates additional reinforcement to the matrix in the form of bound or occluded rubber. Further addition to the filler proportion results in cross over of “filler network percolation threshold”. This actually introduces a flocculated filler network which is susceptible to strain. With a small amount of strain application, this percolated network gets broken down, which is known as the Payne effect [20].

Breakdown of the filler network under a small strain (~0.1% strain) is negligible because the external stress is small enough to rupture the cluster of filler [21]. The filler network starts collapsing with the application of strain. This is evident from the rapid decrease in storage modulus (G’) values with increasing strain [22].

**Figure 4:**
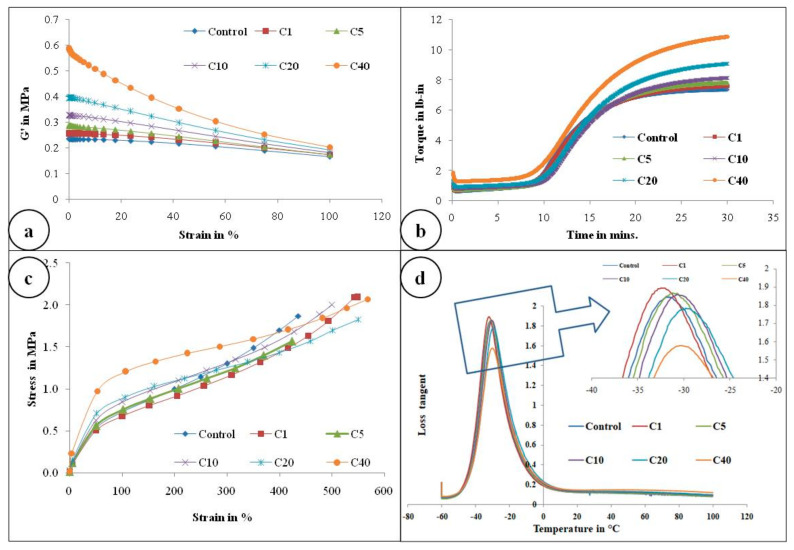
(**a**) Payne effect measurement, G’ (in Pa) against % strain; (**b**) rheological graph of all compounds; (**c**) stress–strain curve of all compounds; (**d**) Plot of tanδ with respect to temperature scan(−60 to +100 °C) of all compounds (Inset: Magnified view of tanδ peak).

Figure 4a represents the storage modulus (G’) against strain (%) of the control gum compound and α-cellulose-containing compounds. It is obvious from the Figure 4a that as filler quantity increases, initial G’ also increases. ∆G’ is proportional to the filler volume fraction. This may be due to the presence of an increasing amount of -OH groups which re-agglomerates further to increase ∆G’.

This phenomenon of re-agglomeration of α-cellulose in the SBR matrix can be well explained by SEM images of cured stocks at different filler volume fractions (Figure 3).

For low filler concentration, isolated filler clusters reinforce the rubber matrix by hydrodynamic effect. The Guth and Gold proposed model is universally accepted to understand hydrodynamic effect for spherical particles. The Guth and Gold equation [19] is:

G’/G’_0_ = 1 + 2.5 *ϕ* + 14.1 *ϕ*^2^ (1)

where, G′ is the initial storage modulus of the filled vulcanizate, G′_0_ is the initial storage modulus of unfilled or gum vulcanizate and *ϕ* is the filler volume fraction. As anisotropic non-spherical filler was used in this work, the modified Guth–Gold equation [23,24] is introduced to explain the rubber–filler interaction [25]:

G’/G’_0_ = 1 + 0.67 f*ϕ* + 1.62 f^2^*ϕ*^2^ (2)

where shape factor f is elucidated as the length to width ratio of cellulose filler aggregates.

**Figure 5:**
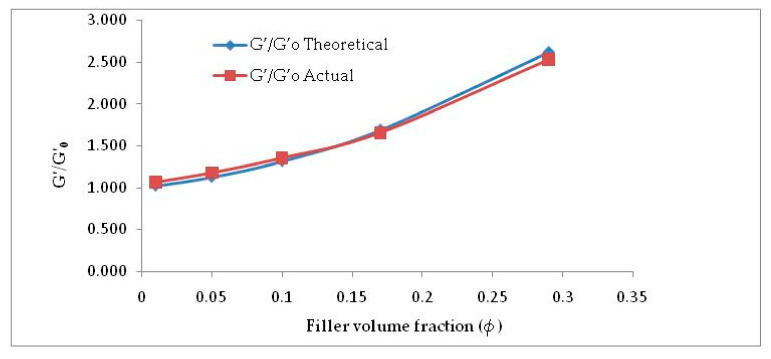
G′/G′_0_ vs. *ϕ* (filler volume fraction)—actual vs. theoretical.

It is perspicuously evident from Figure 5 and Table 2 that considering f = 2.8, Equation (2) is well in agreement with the experimental G′/G′_0_ values. This can be attributed to the incorporation of shape factor as the cellulose particles are found to be non-spherical. The increased filler volume fraction in the rubber matrix develops extensive hydrogen bonding among abundant -OH groups. Increasing α-cellulose dosage in the rubber matrix increases the formation of a larger cluster of filler networks (Figure 3) and showed lesser reinforcement in the E-SBR matrix. Thus, 10% dosage of α-cellulose can be designated as the optimum one with respect to filler networking and dispersion in the matrix.

#### 2.2.3. Vulcametric Characterization of the Composites

Figure 4b and Table 3 illustrate the rheological properties of all the composites. Maximum torque (MH) as well as minimum torque (ML) both increases as α-cellulose is added as filler in the E-SBR matrix. Upsurge of ML values can be attributed to improved rubber–filler interaction. Scorch safety or ts2, which is an important parameter for the processing of rubber compounds, is found to increase up to an addition of 10phr of α-cellulose, but after that, for compounds containing 20 and 40 phr of filler, ts2 of the system decreases. This phenomenon can be ascribed by the fact that hydroxyl (-OH) groups present in the system adsorb ZnO [11] and undergo complex formation. As a result, accelerator activation efficiency diminishes and scorch safety (ts2), consequently, increases. After C10, profound re-agglomeration of α-cellulose forms filler clusters (Figure 3) and, as a result, reinforcement ability of α-cellulose gets reduced in the E-SBR matrix. Poor dispersion and distribution of α-cellulose emanates re-agglomeration of -OH groups in the matrix. Thus, adsorption of ZnO on -OH groups increases effectively (due to increase in α-cellulose in the system). In addition to that, adsorption of the accelerator also takes place on the surface of remaining -OH groups. As a result, ts2 reduces significantly at a higher dosage of α-cellulose. Therefore, from a rheological as well as a processibility point of view, 10 phr addition of α-cellulose in the E-SBR matrix may be considered as optimal filler quantity that invokes optimized rheological behavior and process ability.

#### 2.2.4. Physico-Mechanical and Dynamic Mechanical Characterization of the Compounds

**Figure 6:**
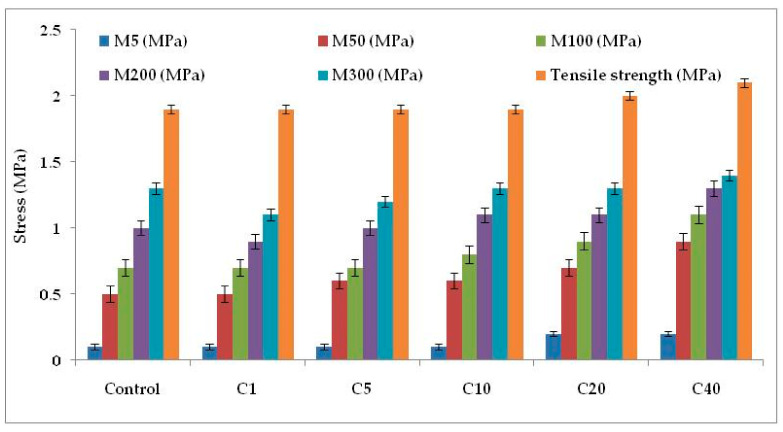
Physico-mechanical properties of the E-SBR-cellulose composites.M5, M50, M100, M200 and M300 denotes modulus at 5% elongation, modulus at 50% elongation, modulus at 100% elongation, modulus at 200% elongation and modulus at 300% elongation, respectively.

Figure 4c and Figure 6 represent the stress–strain behavior of the compounds. Low surface area of α-cellulose instigates agglomeration of filler particles at higher filler loading. The occluded rubber within the agglomerates or aggregates forms a higher modulus rubber shell near the filler surface. Thus the segmental mobility of polymer chains gets seized and the immobilized rubber attached to filler surface leads to an increase in dynamic stiffness. But the polymer chains which are not adsorbed on the filler surface move a distance away from the filler aggregates. Therefore, the static modulus decreases gradually or remains at the same level, as that of the polymer matrix, with increase in filler volume fraction. Similarly, with increase in α-cellulose volume fraction, a significant increase in modulus is not observed. The presences of strong inter and intra molecular H-bond among the -OH groups of low surface area α-cellulose made it difficult for the filler to disperse and distribute throughout the polymer matrix. This phenomenon of re-agglomeration among α-cellulose particles increased with increase in filler volume fraction. Irrespective of that, there is a reinforcement effect of α-cellulose which is evident through the trend analysis of modulus.

Morphological analysis has already enunciated the agglomeration of filler above 10 phr dosage. Thus combining this with ultimate property analysis has clearly established the mentioned dosage as the optimized one.

Therefore, from the above review, it is clear that in the E-SBR matrix, α-cellulose can be used up to 10% level as filler. Compared to gum compound, C10 has shown improvement in ultimate properties. Thus, 10 phr α-cellulose inclusions as filler maybe considered as the optimized dosage with respect to reinforcement in a SBR-based matrix.

Correlation of glass transition temperature (t_g_) with α-cellulose concentration is one of the important parameters that is captured in this article. This positive shift in t_g_ may be explained by the mobility of the polymer chains. SBR has lower entanglements compared to NR. Thus, interaction of filler with SBR reduces structural relaxation time of SBR, resulting in an increase in t_g_ [26]. But the shift is not significant in comparison to the inclusion of filler volume fraction. This phenomenon may be attributed to the fact that the local molecular dynamics of SBR superimposes the interaction between the filler surface and the polymer matrix [26]. Therefore, even if there is a positive shift in t_g_ with increase in filler volume, the change itself is not noteworthy.

Figure 4d and Table 4 clearly depict the peak of tan delta for different composites. This representative figure denotes that, except C20 and C40, other peaks are similar in height. The smaller peak heights of composites having higher filler volume may be attributed to the increased concentration of inter and intra hydrogen bonding among hydroxyl groups present on the α-cellulose surface. This leads to comparatively higher increase in viscous modulus than the storage one. Thus, peak height reduces.

Dynamic mechanical properties of a composite depend on the filler phase characteristic present in the elastomeric matrix. Volume fraction of the filler, filler–polymer interaction, shape of filler particles, and state of agglomeration and mechanical strength of agglomerates are the important parameters that determine the dynamic mechanical properties of an elastomeric composite [27]. The increase in elastic modulus (E’) of a composite is a combination of the above mentioned factors. Here, Table 4 represents the elastic modulus (E′). Increase of E′ may be an indication of polymer–filler interaction but here, in this case, increase of E′ at higher filler volume (particularly for C20 and C40 compound) may be an additional impact of filler–filler interaction (due to presence of a high number of -OH groups on α-cellulose). Nielson et al. has already shown that agglomeration of filler particles largely increases the dynamic modulus of a filled polymer [27]. The filler agglomerates are not wetted by polymers and remain filled with air. This phenomenon induces an increase in the Einstein co-efficient and lowering of the maximum packing fraction (m) of the filler agglomerate [27].

The lowering of *ϕ*_m_ may be characterized by the Krieger-Dougherty equation [28]. The presence of strong interaction of surface -OH groups might result in an increase in E′ with higher filler volume. From the Krieger-Dougherty equation, it is very much evident that an increase in filler volume fraction results in the increase in matrix viscosity. *ϕ*_m_ of filler agglomerates proportionately lowers viscosity of the medium.

**Figure 7:**
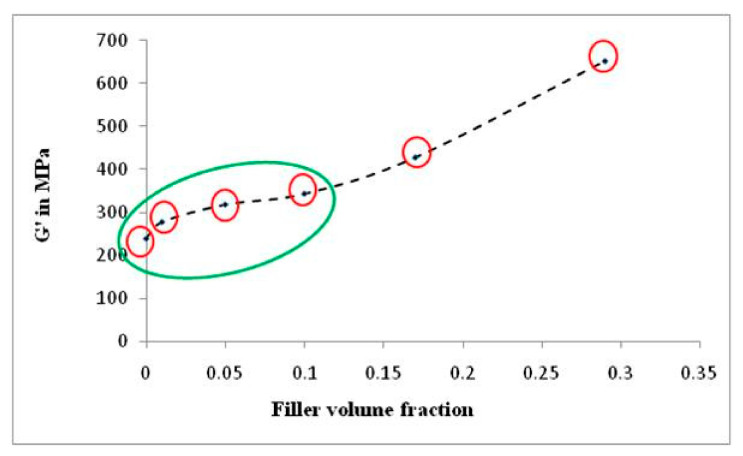
Plot of dynamic modulus (G′) vs. filler volume fraction (*ϕ*) in shear mode.

To reinforce the occurrence of re-agglomeration of α-cellulose aggregates at a higher filler volume fraction (C20 and C40), dynamic modulus (G′) at shear mode is plotted against filler volume fraction (*ϕ*) in Figure 7. The figure demonstrates that G’ increases abruptly as we move from C10 to C20 and further. This indicates that, as we move towards higher α-cellulose dosage, simultaneous agglomeration of filler was obtained which results in lowering of *ϕ*_m_. This representative graph clearly depicts the optimum filler (α-cellulose) level in the C10 compound. Therefore, the C10 compound can be designated as the optimized one (with respect to dispersion and distribution of filler in polymer matrix), where storage modulus and loss tangent both has increased in the tune of ~20% from the control one.

#### 2.2.5. Rubber–Filler Interaction

Adsorption of elastomeric chains within the filler aggregates invokes reinforcement to the matrix. Researchers have employed several methods to ascertain rubber–filler interaction. Lorenz and Park equation is one of the universally accepted equations which are applied here to understand the reinforcement potency of α-cellulose in the composite through rubber–filler interaction [29]. The toluene-based swelling test is engaged to measure the interaction. Free spaces present in the matrix are occupied by toluene. Thus, cross-linked sites are proportional to the rubber–filler bonding and inversely related to the amount of toluene absorption [30].

Table 5 represents the rubber–filler interaction pertaining to the ratio of Q_f_ and Q_g_. Elastomeric cohesion with filler aggregates reinforces the matrix. As a result, occluded rubber is developed and, consequently, cross-linking sites get generated. When a composite is immersed in toluene, solvent uptake by sample proportionately decreases with an increase in cross-linked sites induced by the bound rubber. Thus, Q_f_/Q_g_ is proportional to the amount of solvent uptake by the composite. As a result, the higher the Q_f_/Q_g_, the lower the polymer–filler interaction. Here, C10 has the least Q_f_/Q_g_, which demonstrates that the composite with 10 phr filler contains the highest polymer–filler interaction amongst all the composites. Although C20 and C40 compounds contain higher levels of α-cellulose, the 10 phr dosage of α-cellulose has emerged as the optimal one in the E-SBR-α-cellulose composite.

The studies so far have established that 10 phr α-cellulose can be considered as the optimized dosage in E-SBR composites. With this background, a typical formulation having synthetic filler (Silica) is evaluated against 10 phr substitution with natural filler (α-cellulose). Table 6 denotes the evaluated recipe and corresponding properties.

**Figure 8:**
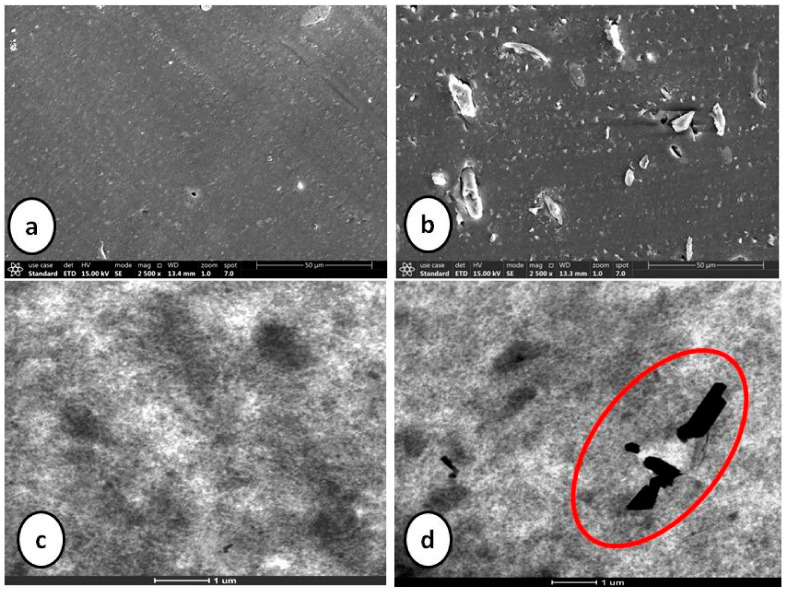
SEM (**a**,**b**) and TEM (**c**,**d**) images of silica-filled control and experimental compounds.

Table 6 shows the mapping of rheological, physico-mechanical and dynamic mechanical properties of composite containing silica as sole filler against the composite containing 10 phr silica substituted by cellulose. Introduction of α-cellulose increases the abundance of hydroxyl groups (due to its presence on cellulose surface) which results in an increase of the cure rate. Additionally, more adsorption of accelerator results in reduced scorch safety with simultaneous increase in CRI of the substituted composite. Physico-mechanical properties of both the composites are close enough with increased amplitude of storage modulus. Agglomeration of silica particles may be the enunciated as the attributed cause. Introduction of α-cellulose in the experimental compound has increased the inter-aggregate distance among silica particles, which has improved the dispersion as well as distribution of silica in the polymer matrix (Figure 8). Thus, it can be concluded that silica may be substituted by 10 phr of α-cellulose in an E-SBR composite, as this can be designated as the morphologically and physico-mechanically optimized dosage.

## 3. Materials and Methods

### 3.1. Chemicals

E-SBR (SBR 1502) with 23.9% styrene (measured as per ISO 21,561 method) was procured from Asahi Kasai, Tokyo, Japan. Ash content and volatile matter of this E-SBR is 0.13% (ASTM D5667) and 0.23% (ASTM D5668), respectively. Mixed organic acid, soap content and acetone extraction were also measured in the laboratory as per ASTM D5774 method. ML (1 + 4) @ 100 °C of the E-SBR (massed) was 52.9 MU. Other compounding ingredients used were as follows: Silica (Specialties silica ltd., Alwar, India), zinc oxide (POCL enterprises ltd., Pondicherry, India), stearic acid (Godrej industries ltd., Gujarat, India), α-cellulose (Suhal cellulose llp, Kolkata, India), TBBS (N-tertiarybutyl-2-benzothiazolesulfenamide) (National organic chemicals industries ltd., Mumbai, India), soluble sulfur (The standard chemical co. pvt. Ltd., Chennai, India).

### 3.2. Characterization of α-Cellulose

α-cellulose mentioned in this work is used as received from supplier. It is an odorless, off-white free flowing powder with the mentioned chemical characteristics: Nitrogen surface area of 1.60 m^2^/g (ASTM D1993); 4.19% of heat loss at 70 °C (ASTM D4571); ash content of 0.33% (ASTM D4574); pour density of 356.2 kg/m^3^(ASTM D1513) with 4.05 pH (ASTM D6739). Silica used in this work had the following characteristics—5.5% heat loss at 105 °C for 2 h (ASTM D 6738); pH of 6.3 (ASTM D 6739); 4.5% ignition loss at 950 °C for 2 h and nitrogen surface area of 170 cm^2^/gram.

In order to detect the functional groups associated to α-cellulose, Fourier transform infrared (FTIR) spectroscopy is employed. α-cellulose mixed with spectroscopy grade potassium bromide (KBr) powder underwent scan ranging from 400 to 4000 cm^−1^ at a resolution of 4 cm^−1^ in FTIR 2000 system from Perkin-Elmer (Norwalk, CT, USA).

Thermal stability of α-cellulose is analyzed by Thermo gravimetric analysis (TGA) using Pyris-1 TG analyzer of Perkin-Elmer (Shelton, CT, USA). The sample is scanned at a heating rate of 10 °C/min. Initially nitrogen is used as the heating medium to increase temperature from room temperature to 580 °C and then shifted to oxygen and further heated up to 850 °C.

Thermal reactivity of α-cellulose is studied by using differential scanning calorimeter (DSC) with diamond DSC system (Perkin Elmer, Waltham, MA, USA), at a heating rate of 10 °C/min.

#### 3.2.1. SEM Topography 

Surface topography of α-cellulose and SBR composites are captured by field emission scanning electron microscope (ApreoS, FEI, Hillsboro, OR, USA). It is operated in secondary electron (SE) modeat an accelerating voltage of 20 kV after coating the samples with platinum using a sputter coaterQ150T ES (Quorum Technologies, Lewes, UK)

#### 3.2.2. TEM Imaging

Dispersion of α-cellulose in SBR composites is further confirmed by taking images under HRTEM, 200 kvTalos-S (FEI, Hillsboro, OR, USA). All the images are captured at fixed magnification. Thin specimen of around 100 nm is prepared by ultra-microtomy (Leica Ultracut UCT) at −100 °C to acquire high resolution images.

### 3.3. Preparation of Composites

As per Table 1, a gum rubber compound is selected as control formulation. In experimental compounds, α-cellulose is used as filler at different dosages starting from 1 to 40 phr, to understand its reinforcement effect in E-SBR-based formulation and to optimize its dosage in similar compounding recipes. Master, re-pass and final mixing of compounds are done in a laboratory-scale Haake mixer (Thermo-fischer, Dreieich, Germany) of 300 cc chamber volume, maintaining fill factors of 0.72, 0.75 and 0.75, respectively. This mixer is two-wing rotor based. The temperature control unit (TCU) is set at 90 °C for master and re-pass, and at 70 °C for final compound mixing. For master and re-pass, rotor speed was maintained at 50–55 rpm and the discharge temperature of the batch is maintained at 160 °C, whereas for final compound mixing, rotor speed was maintained at 30 rpm and batch discharge temperature was in between 95 and 100 °C. During master compound mixing, initially at 0 s, rubber was introduced to mixer and carbon black along with all master chemicals were added after 15 s. The total master mixing cycle was for 225 s. Repass and final mixing cycle was of 180 s each.

### 3.4. Measurement of Payne Effect

Payne effect [21] indicates the dispersion of filler in rubber matrix. Storage modulus (G′) is measured in the rubber process analyzer (model RPA 2000, Alpha technologies, Akron, OH, USA) under a strain sweep from low strain (0.1%) to high strain (100%). Preheating is done for ~5 grams of sample at 50 °C within the closed cavity of the equipment for 5 min, with 0.1% strain at 1.67 Hz frequency to release the stress developed during the closing time of the instrument. Strain sweep from 0.1% to 100% was applied at a constant temperature of 50 °C and 1.67 Hz frequency.

Payne effect is calculated by using Equation (3):

(3) $$\Delta G^{\prime }=G^{\prime }\;\mathrm{at}\;0.1\%\;\mathrm{strain}\;-\;G^{\prime }\;\mathrm{at}\;100\%\;\mathrm{strain}\;$$

### 3.5. Determination of Cured Characteristic

Rheological properties of the compounds are ascertained using a moving die rheometer (model Premier MDR, Alpha technologies, Akron, OH, USA). The tests are performed at 145 °C for 60 min using 0.5° arc in RPA. Rheological properties are generated by complying with ASTM D5289. Optimum cure times (tc90) of the compounds are calculated from the graph by using Equation (4).

(4) $$\mathrm{tc}90\;\left(\min\right)=0.9\;\left(\mathrm{MH}-\mathrm{ML}\right)\;+\;\mathrm{ML}$$

where, MH and ML are corresponding maximum and minimum torque respectively.

Cure rate index (CRI) was determined by using Equation (5):

(5) $$\mathrm{CRI}\;=\;\frac{100}{\left(\mathrm{tc}90-\mathrm{ts}2\right)}\;\min^{-1}$$

where ts2 corresponds to the time to raise 2 unit torques above the minimum torque.

### 3.6. Preparation of Molded Slabs and Measurement of Physico-Mechanical Properties

Complying with ASTM D3182, rubber compounds are molded in an electrically heated hydraulic press (Hind hydraulics, New Delhi, India) using compression molding technique. Tensile slabs are cured at 160 °C and time corresponding to 2×tc90 min under 150 kg/cm^2^ molding pressure. The dimensions of the cured slabs were 15 cm × 15 cm × 2 mm (length × width × thickness).

Tensile properties are determined by punching specimens from cured slabs. Dumbbell shaped tensile samples are cut and tested as per ASTM D412 using Z010 universal testing machine (UTM) procured from Zwick (Ulm, Germany).

### 3.7. Measurement of Dynamic Mechanical Properties

Storage modulus (E′), loss modulus (E″) and loss tangent (tan δ) are determined at 30, 70 and 100 °C, respectively, using dynamic mechanical testing. Tension–relaxation mode is employed by using the visco-analyzer VA4000 Metravib, R.D.S, France. In this experiment, a sample having the thickness of 2.7 ± 0.2 mm is tested under a temperature scan from −60 to +100 °C. Frequency and strain values are 11 Hz and 1%, respectively. Loss tangent at different temperatures are measured as per the relation of

$$\mathrm{Tan}\;\delta \;=\;\frac{E^{\prime \prime }}{E^{\prime }}$$

### 3.8. Measurement of Rubber-Filler Interaction

Cured specimens of 30 × 5 × 2 mm are immersed in toluene and, after specific intervals, samples are taken out of toluene to measure the weight. It was observed that after 48 h at 25 °C, the specimens achieved equilibrium weight [29]. Then the following Equation (6) is employed to determine the solvent uptake (Q):

(6) $$\mathrm{Solvent}\;\mathrm{uptake}\;(Q)\;=\;\frac{W_{\mathrm{eq}}-W_{0}}{W_{0}}\;\times \;100$$

where, W_eq_ is the equilibrium weight of the sample and W_0_ is the initial weight of the solvent.

Then, the rubber-filler interaction is measured by Q_f_/Q_g_ ratio [30], where f and g stand for filled and gum vulcanizate, respectively.

## 4. Conclusions

The chemical characterization and morphological uniqueness of α-cellulose has generated immense enthusiasm to assess its behavior as filler in a rubber matrix. In this article, E-SBR is chosen due to its lower gum strength where the reinforcing ability of filler can be well understood. Microscopic analysis by SEM and TEM are performed to understand the morphological threshold. Both studies revealed that C10 compound with 10 phr filler can be designated as the optimal one with respect to dispersion and distribution in E-SBR matrix. Rheological and physico-mechanical evaluation of the composites also reinforces C10 as the optimal composite. Visco-elastic, flow and dynamic mechanical behavior of the composites clearly depicts an increasing trend with increased volume fraction of filler. These characteristic features have been further analyzed by interpreting the trend of dynamic modulus and its slope with respect to filler volume. This is to understand and estimate filler agglomeration threshold and, further to this, its impact on the visco-elastic and dynamic behavior. The previous observations pertaining to C10 compound as the optimized one were further established. Accordingly, the reinforcement study using the Guth and Gold equation and the investigation of the rubber–filler interaction clearly substantiates that C10 is the optimal E-SBR-α-cellulose composite amongst all. This optimized dosage is further examined by substituting silica with α-cellulose. Rheological, physico-mechanical and dynamic mechanical properties clearly establish the reinforcing ability and silica replacement capability of α-cellulose.

## Figures and Tables

**Table 1:** Formulation (in phr) used for α-cellulose-E-SBR composite. Here ‘phr’ stands for parts per hundred of rubber.

	Control	C1	C5	C10	C20	C40
SBR	100	100	100	100	100	100
ZnO	3	3	3	3	3	3
Stearic acid	1	1	1	1	1	1
α-cellulose	0	1	5	10	20	40
Sulfur	1.75	1.75	1.75	1.75	1.75	1.75
TBBS	1	1	1	1	1	1

ZnO: Zinc oxide; TBBS: *N*-tertiarybutyl-2-benzothiazolesulfenamide.

**Table 2:** Actual and theoretical values of G′/G’_0_ at different filler volume fractions.

*ϕ*	*ϕ* ^2^	G′/G′_0_ Theoretical	G′/G′_0_ Actual
0.01	0.0001	1.020	1.066
0.05	0.0025	1.126	1.180
0.1	0.01	1.315	1.356
0.17	0.0289	1.686	1.658
0.29	0.0841	2.612	2.531

**Table 3:** Rheological properties of the composites.

Curing Parameters						
Parameters	Control	C1	C5	C10	C20	C40
ML(lb.in)	0.62	0.66	0.71	0.79	0.93	1.28
MH(lb.in)	7.36	7.58	7.82	8.1	9.08	10.86
MH–ML	6.74	6.92	7.11	7.31	8.15	9.58
ts2 (min)	10.78	11.1	11.57	12.04	11.87	11.03
tc50 (min)	12.31	12.82	13.35	14.02	14.24	14.16
tc90 (min)	18.95	19.99	20.36	21.34	22.06	22.67
CRI (min^−1^)	12.2	11.2	11.4	10.8	9.8	8.6

**Table 4:** Dynamic-mechanical properties of the compounds.

Dynamic Mechanical Properties						
**30 °C**						
	**Control**	**C1**	**C5**	**C10**	**C20**	**C40**
E′ (MPa)	2.79	2.85	3.07	3.46	4.18	6.43
E″ (MPa)	0.28	0.3	0.32	0.39	0.48	0.85
Tanδ	0.101	0.104	0.104	0.113	0.115	0.132
**70 °C**						
	**Control**	**C1**	**C5**	**C10**	**C20**	**C40**
E′ (MPa)	2.56	2.61	2.78	3.09	3.65	5.32
E″ (MPa)	0.21	0.23	0.24	0.3	0.36	0.65
Tanδ	0.082	0.087	0.087	0.098	0.1	0.123
**100 °C**						
	**Control**	**C1**	**C5**	**C10**	**C20**	**C40**
E′ (MPa)	2.28	2.38	2.63	2.8	3.38	4.71
E″ (MPa)	0.14	0.16	0.17	0.21	0.25	0.46
Tanδ	0.062	0.065	0.064	0.075	0.075	0.097

**Table 5:** Rubber-filler interaction (Q_f_/Q_g_).

Compound	C1	C5	C10	C20	C40
Q_f_/Q_g_	1.02	1.09	0.91	0.97	0.95

**Table 6:** Formulation, rheological, physico-mechanical and dynamic properties of silica-containing composites.

Ingredients	Unit	Control		Experimental	
SBR	phr	100		100	
Zinc oxide	phr	3		3	
Stearic acid	phr	1		1	
Silica	phr	50		40	
α-cellulose	phr	0		10	
Sulfur	phr	1.75		1.75	
TBBS	phr	1		1	
**Rheological Properties**					
**Parameters**			**Unit**	**Control**	**Experimental**
Minimum torque		ML	lb.in	1.70	1.51
Maximum torque		MH	lb.in	19.21	18.95
Max-min. Torque		MH-ML	lb.in	17.51	17.44
Time corresponding to two units torque rise		ts2	min	4.84	4.29
Time corresponding to 40% cure		tc40	min	6.19	5.42
Time corresponding to 90% cure		tc90	min	10.18	9.00
Cure Rate Index		CRI	min^−1^	18.73	21.23
**Physico-mechanical properties**					
**Parameters**			**Unit**	**Control**	**Experimental**
Modulus at 5% elongation		M5	MPa	0.5	0.4
Modulus at 20% elongation		M20	MPa	1	1
Modulus at 50% elongation		M50	MPa	1.5	1.5
Modulus at 100% elongation		M100	MPa	2.3	2.2
Modulus at 200% elongation		M200	MPa	5.2	5
Modulus at 300% elongation		M300	MPa	9.6	9.4
Tensile strength		TS	MPa	18.4	18.2
Elongation at break		EB	%	465	472
Hardness			Shore A	68	67
**Dynamic-mechanical properties**					
**Parameters**			**Unit**	**Control**	**Experimental**
Storage modulus at 30 °C		E′ at 30 °C	MPa	17.21	14.15
Storage modulus at 70 °C		E′ at 70 °C	MPa	11.47	9.96
Storage modulus at 100 °C		E′ at 100 °C	MPa	9.19	8.38
Loss modulus at 30 °C		E″ at 30 °C	MPa	2.98	2.23
Loss modulus at 70 °C		E′ at 70 °C	MPa	1.77	1.33
Loss modulus at 100 °C		E′ at 100 °C	MPa	1.23	0.90
Loss tangent at 30 °C		Tanδ at 30 °C		0.173	0.158
Loss tangent at 70 °C		Tanδ at 70 °C		0.154	0.134
Loss tangent at 100 °C		Tanδ at 100 °C		0.134	0.107

## Data Availability

This study doesn’t report any supporting data for this manuscript.

## Sample Availability

Samples of the compounds are available from the authors.
